# The Combined Effect of Green Tea, Saffron, Resveratrol, and Citicoline against Neurodegeneration Induced by Oxidative Stress in an *In Vitro* Model of Cognitive Decline

**DOI:** 10.1155/2024/7465045

**Published:** 2024-10-01

**Authors:** Simone Mulè, Sara Ferrari, Giorgia Rosso, Rebecca Galla, Stefania Battaglia, Valeria Curti, Claudio Molinari, Francesca Uberti

**Affiliations:** ^1^ Department for Sustainable Development and Ecological Transition Laboratory of Physiology, Via Sant Eusebio 37, Vercelli (VC) 13100, Italy; ^2^ Noivita S.r.l.s. UPO Spin-Off of University of Eastern Piedmont, Via Solaroli 17, Novara (NO) 28100, Italy; ^3^ R&D Department Kolinpharma S.p.A., Corso Europa 5, Lainate (MI) 20045, Italy

## Abstract

During ageing, the brain is vulnerable to a growing imbalance of the antioxidant defence system, resulting in increased oxidative stress. This condition may be mainly responsible for cognitive decline, resulting in synaptic transmission disruptions and the onset of neuronal dysfunction. In this context, developing efficient preventive and therapeutic strategies against increased oxidative stress and decreased antioxidant defence mechanisms should be considered a public health priority to promote healthy ageing. Therefore, the current study explored the benefits of a novel combination of green tea, saffron, trans-Reveratrol, and citicoline, called MIX, on improving intracellular processes to ameliorate the mechanisms linked to cognitive decline under oxidative stress conditions. First, the ability of MIX to cross the blood-brain barrier (BBB) was evaluated in an *in vitro* model, analysing TEER value and the specific tight junctions; second, the CCF-STTG1 cell line was pretreated with 200 *µ*M H_2_O_2_ for 30 min to explore the effects of the single active compounds and their combination under oxidative stress conditions. Our results demonstrated for the first time the synergistic effects of the new combination to improve the absorption rate of individual agents through the BBB and maintain its integrity. Subsequently, further research was done to assess the positive role of the combination to counteract oxidative damage; as expected, MIX restored the neurodegenerative state activated by 200 *µ*M H_2_O_2_, reducing mitochondrial damage, and improving survival pathways. Additionally, MIX acted as a regulator of both cellular energy metabolism and apoptosis, reducing the inflammatory state activated by oxidative stress. Finally, MIX can balance neurotrophin production to prevent mitochondrial disruption. In conclusion, MIX counteracted the adverse effects of brain oxidative stress, suggesting that this new proposed formulation prevents the molecular mechanisms underlying the onset of cognitive decline, even in support of conventional therapy.

## 1. Introduction

The decline of mental functions is a growing public health issue. It consists of a deterioration in cognitive abilities due to several factors, including ageing [1]. Ageing is defined as a progressive accumulation of physiological changes related to advancing age, which may increase the onset of diseases. One of the districts most susceptible to the physiological ageing process is the brain, resulting in the onset of loss of brain function that may lead to neurodegenerative diseases [2]. Different mechanisms may accelerate brain ageing and transform it into a pathological process. Among these, oxidative stress is one of the main drivers. The formation of reactive oxygen species (ROS) in the brain causes damage to DNA, proteins, and lipids, generating a pro-inflammatory state that leads to the degeneration of pathways involved in cognitive function [3]. The brain is especially vulnerable to oxidative injury due to its high oxygen consumption, high concentration of transition metals (e.g., iron or copper), and abundance of polyunsaturated fatty acids susceptible to oxidation. Neurons can degenerate due to prooxidant buildup and weakened antioxidant defences. The most affected and vulnerable brain areas are the hippocampus, substantia nigra, amygdala, and frontal cortex, namely, those regions mainly involved in the memory storage mechanism [3, 4, 5]. In this context, developing efficient preventive and therapeutic strategies against the pathological ageing process related to cognitive function decline should be considered a public health priority [6]. Numerous investigations on the biological effects of vitamins, polyphenols, oligo-elements, and *ω*−3 polyunsaturated fatty acids have emerged as promising therapeutic agents to counteract the neurodegenerative pathways associated with cognitive impairment [7]. Secondary plant metabolites, such as flavonoids and diarylheptanoids, have exhibited antioxidant properties that could be used to treat oxidative stress-induced neurological disorders [8, 9]. Some active substances belonging to this class of molecules can be used to formulate functional food supplements, which could be useful in treating and preventing neurodegenerative disorders such as cognitive decline [7, 10]. Nutraceutical interventions can potentially decrease prooxidant levels in the brain by modulating mitochondrial stress, apoptotic factors, the free radical scavenging system, and neurotrophic factors. These actions could inhibit pro-inflammatory and pro-amyloidogenic processes, thereby delaying neurodegeneration [7].

In this context, green tea is a promising extract [11], which its polyphenols content reaches 70% and includes epigallocatechin−3-gallate (EGCG, predominant catechin), epigallocatechin (EGC), gallocatechin, epicatechin−3-gallate (ECG), and epicatechin (EC) [12]. From a biological point of view, catechins are the most representative active molecules in Green Tea dry extract; among these, the most significant is EGCG [13]. Several scientific research studies have recently explored the pharmacological activities of Green Tea catechins in the inflammatory cascade process, oxidative damage, cellular transcription, and transduction pathways in several targets. Indeed, EGCG has shown its ability to counteract pro-apoptotic protein levels, such as caspase−3 and Bax, in a neuroblastoma cellular model transfected with human APP695. In this experimental condition, EGCG treatment attenuated the *β* amyloid protein generation, acting as a PPAR*γ* agonist and suppressing the transcription and translation of BACE1, a protein responsible for APP cleavage [14]. Moreover, EGCG exerts its neuroprotective effect thanks to its anti-inflammatory action. EGCG significantly reduces, on the one hand, the levels of Iba1 and its co-localization with Caspase−1, which is responsible for the activation of Interleukin 1 in the microglia. On the other hand, it interferes with inflammasome activation through the toll-like receptor 4 (TLR4)/NF-*κ*B pathway [15].

Another extract with supporting evidence in counteracting cognitive decline and related impairments, such as sleep and mood disorders, is saffron (*Crocus sativus* L.), a plant in the *Iridaceae* family, native to Asia Minor and Mediterranean countries. Generally, the main compounds of the saffron dry extract are crocin (29–300 mg/g), safranal (0.06–0.29 mg/g), picrocrocin (2.18–6.15 mg/g), and non-glycosylated C20-dicarboxylic acids [16]. Recent studies have examined the potential of saffron dry extract to mitigate cognitive decline, control glutamate signalling, lessen oxidative stress, and alter tau and A*β* aggregation in preclinical and clinical settings [17, 18, 19]. Specifically, in an animal model of cognitive impairment due to tramadol toxicity, crocin (30 mg/kg) administered orally for 28 consecutive days improved learning and memory in treated rats by reducing the apoptotic cells in the hippocampus [20]. Several clinical studies demonstrated the efficacy of saffron both on dementia and on mood and sleep quality. Akhondzadeh et al. [21] have shown that a dosage of 30 mg/day of saffron dry extract is able in 16 weeks to significantly improve cognitive performance staged by completing the ADAS-cog and CDR-SB questionnaires compared to placebo. Another study highlighted that the cognitive improvement obtained in 22 weeks, thanks to the administration of saffron dry extract, is comparable to the efficacy of donepezil treatment [22]. Comparably, Farokhnia et al. [23] showed that the effects of memantine medication on cognitive impairment in patients with moderate to severe AD disease were equivalent to those of saffron.

Numerous experimental and clinical models have investigated resveratrol's antioxidant, anti-inflammatory, and neuroprotective properties. This well-known phytoalexin may be found in blackberries, wine, black grapes, peanuts, and chocolate. Resveratrol can indirectly mitigate oxidative stress by affecting PGC−1 *α* through its impact on SIRT1 [24]. In mice, pre-treatment with resveratrol increases SIRT1 levels, regulating the expression of mediators of inflammation and apoptosis, including IL−1*β*, TNF-*α*, caspase 3, Bax, and Bcl2. Additionally, SIRT1 controls the expression of BDNF by an epigenetic mechanism linked to neuronal renewal and synaptic plasticity [25]. Resveratrol decreases the activity of enzymes involved in oxidative stress development by directly reducing free radical production in tissues and upregulating the expression of oxidative stress-inducing proteins, such as glycogen synthase kinase 3*β* (GSK-3*β*). In some clinical trials, Resveratrol has been noted to reduce *β*-amyloid (A*β*) protein levels and improve brain volume in Alzheimer's patients, preventing cognitive decline. Antioxidant and anti-inflammatory properties may be linked to preventing neurodegenerative illnesses [26].

Furthermore, resveratrol can also represent great interest in managing cognitive decline thanks to its demonstrated action in improving cerebral blood flow. Ageing causes artery stiffness, affecting vasodilation and compromising cerebral perfusion and cerebrovascular responsiveness [27]. Another post-menopausal clinical study showed that taking trans-resveratrol for 12 months improves processing speed and cognitive flexibility, impacting overall cognition. The systolic, diastolic, and mean flow rate study has shown significant resveratrol efficacy. Therefore, the ability of resveratrol to improve cognitive performance is due to its ability to improve brain perfusion [28]. Resveratrol increases endothelial nitric oxide synthase (eNOS activity), increasing NO production and improving its bioavailability at the vascular level [29]. Finally, citicoline is a substance whose use is widely known in memory and cognitive function improvement. Citicoline or cytidine diphosphate (CDP)-choline is composed of two different molecules: cytidine and choline. It promotes acetylcholine biosynthesis and tyrosine hydroxylase activity, a key enzyme for synthesising dopamine. In addition, citicoline participates in the biosynthesis of membrane phospholipids, resulting in the fundamental integrity of cell membranes [30, 31]. Clinical studies have shown its effectiveness in several central nervous system (CNS) disorders, including cognitive decline and dementia [32, 33, 34]. Treatment with 500 mg of citicoline per day for 2 weeks has been shown to improve human vigilance, visual working memory, and oxidative stress markers compared to placebo. Citicoline has also significantly decreased serum malondialdehyde (MDA) levels in these patients [33]. Citicoline has also been studied for its anti-inflammatory and neuroinflammatory activities as it can reduce the secretions of cytokines/chemokines MIP-1 *α*, TNF*α*, IL-1*β*, monocyte chemoattractant protein-1 (MCP-1), IL-6, regulated upon activation, normal T cell expressed and presumably secreted (RANTES) and the anti-inflammatory cytokine, interleukin-10 (IL-10) [35, 36].

In light of the substantial scientific evidence that these substances have been utilised in clinical settings to counteract the mechanisms underlying cognitive decline, the purpose of this study is to examine the molecular mechanisms by which a novel combination of green tea, saffron, trans-resveratrol, and citicoline might enhance cerebral function in a neurodegeneration model subjected to moderate oxidative stress.

## 2. Materials and Methods

### 2.1. Agents Preparations

Saffron (Saffron D.E. 0.3% used in a range of concentration from 25 to 200 *µ*M) [37, 38], trans-Resveratrol (called trans-R used in a range of concentration from 1 to 10 *µ*M) [39, 40], green tea (*Camellia sinensis* L., Kuntze, 60% catechins- 40% EGCG used in a range of concentration from 25 to 200 *µ*M) [41, 42], and citicoline (Kyowa, used at 100 *µ*M) [43] donated by Kolinpharma S.p.A (Kolinpharma S.p.A., Lainate, Milan, Italy) were examined separately or combined after preparing them directly in Dulbecco's Modified Eagle Medium (DMEM, Merck Life, Milan, Italy). All substances were precisely administered to the cells at a molar concentration of the active ingredient used to titrate the raw material. Consequently, the concentration references are focused on EGCG for green tea and crocin for saffron. In particular, green tea was extracted from *C. sinensis* L. leaves using water and ethyl acetate. Then, green tea was analysed for total catechins and EGCG content, resulting in ~60% and 40%, respectively. Conversely, Saffron was derived from the saffron stigma through an extraction technique that relied on aqueous and alcoholic phases while operating under specific pressure and temperature conditions. This extract underwent titration based on its active components, revealing ~0.34% safranal, 0.37% picrocrocrocins, and 0.43% crocins. To summarise, the ratio of the drug extract is around 1 : 10–20 (saffron stigma: Satiereal powder). The 200 *µ*M H_2_O_2_ (Merck Life Science, Milan, Italy) stimulation was also made in the same medium as the other agents [44]. Brain-derived neurotrophic factor (BDNF) (Merck Life Science, Milan, Italy) was prepared at a starting concentration of 5 *µ*g/mL in sterile phosphate-buffered saline (PBS1x) and diluted in DMEM without red phenol to have a concentration of 10 ng/mL in a stimulation well [45].

### 2.2. Cell Culture

The biological effects of green tea, saffron, trans-R, and citicoline were studied using the human astrocyte cell line CCF-STTG1 from the American Type Culture Collection (ATCC, Manassas, VA, USA). These cells were derived from the brain of a 68-year-old astrocytoma patient and were cultured in flasks in Roswell Park Memorial Institute medium (RPMI, Merck Life Science, Rome, Italy) supplemented with 10% FBS; Merck Life Science, Milan, Italy), 2 mM HEPES (Merck Life Science, Milan, Italy), 2 mM L-Glutamine (Merck Life Science, Milan, Italy) and 1% penicillin-streptomycin (P/S, Merck Life Science, Rome, Italy) and maintained in an incubator at 37°C with 95% humidity and 5% CO_2_. Every 48 hr, the culture media were replaced, and the cell development was routinely checked. The cells were used for experiments at passage 2 after reaching 75%–85% confluence [46]. For the experiments, 1 × 10^4^ cells were plated on a 96-well plate to study cell viability by the 3-(4,5-Dimethylthiazol-2-yl)-2,5-diphenyltetrazolium bromide (MTT) test and ROS production by the colourimetric test; 1 × 10^6^ cells were plated on a 6-well plate to quantify TNF*α*, interleukin-2 (IL-2), BDNF, and to investigate the molecular pathways involved in neuroprotection during oxidative stress condition by ELISA Kit [44].

The human umbilical vein endothelial cells (HUVEC) purchased from ATCC (Manassas, VA, USA) were cultured in 0.1% gelatin-coated flask with Endothelial Growth Medium-2 (EGM-2) containing 2% FBS, 0.04% hydrocortisone, 0.4% hFGF-B, 0.1% VEGF, 0.1% R3-IGF-1, 0.1% ascorbic acid, 0.1% hEGF, 0.1% GA-1000, 0.1% heparin (all from Lonza, Walkersville, MD, USA) [47]. Before being utilised in the research, the HUVEC cells were cultured till passages 3 through 6 at 37°C in a humidified environment of 95% air with 5% CO_2_ [48]. Thus, to perform *in vitro* blood-brain barrier (BBB), 1 × 10^5^ HUVEC cells/cm^2^ were plated in the apical compartment of 6.5 mm Transwells® with a polyester membrane with a pore size of 0.4 *μ*m [49].

### 2.3. Experimental Protocol

Green tea, saffron, trans-R, and citicoline alone and in combination were used to stimulate the astrocyte cells to analyse the mechanisms linked to cognitive decline and neurodegeneration under oxidative stress conditions. In the first set of experiments, a dose-response study on cell viability of CCF-STTG1 cells was performed with green tea (ranging from 25 to 200 *µ*M) [41, 42], saffron (ranging from 25 to 200 *µ*M) [37, 38] and trans-R (ranging from 1 to 10 *µ*M) [39, 40], to select the optimal concentration of these three agents after 24 hr of stimulations. Then, the beneficial concentration of green tea, saffron, and trans-R were tested on CCF-STTG1 cells alone and in combination with citicoline 100 *µ*M [43] to analyse cell viability, BBB permeability (from 2 to 24 hr) and BBB integrity with transepithelial electrical resistance (TEER) value and the specific tight junction (TJ). In a second set of experiments, the CCF-STTG1 cell line was pretreated with 200 *µ*M H_2_O_2_ for 30 min to explore the effects of the combination under oxidative stress conditions compared to the single agents [44] by analysing cell viability, ROS, and NO productions. In addition, inflammatory biomarkers (TNF*α* and IL-2), BDNF production, and intracellular apoptotic and neurodegenerative markers activated were also analysed by ELISA kit.

### 2.4. Cell Viability Test

A 96-well plate was used to measure cell viability of CCF-STTG1 cells after each stimulation using MTT-based *In Vitro* Toxicology Assay Kit (Merck Life Science, Milan, Italy), as reported in the literature [50]. After stimulating cells, 1% MTT dye was incubated at 37°C in an incubator with 5% CO_2_ and 95% humidity, and purple formazan crystals were dissolved in MTT solubilisation solution after 2 hr. In addition, during the quantification, the background interference due to the extract was considered by making an empty sample (without cells but with the treatment), which was subtracted. To assess cell viability, absorbance at 570 nm with 690 nm correction was measured using a spectrometer (Infinite 200 Pro MPlex, Tecan, Männedorf, Switzerland) and compared to the control (0 line, which is 100% viable cells) and expressed in percentage.

### 2.5. BBB *In Vitro* Model

The CCF-STTG1 cell line was cocultured with the HUVEC cell line according to the protocol described in the literature [49]. Initially, 4 × 10^4^ cells/cm^2^ were placed on the basolateral side of the inverted 6.5 mm Transwells® with a polyester membrane having 0.4 *μ*m pore size (Corning Costar, Merck Life Science, Rome, Italy) and allowed to adhere for 4 hr. After that, the cells were allowed to proliferate in Transwells® for 48 hr in their typical orientation. Following a 48-hr incubation period, 1 × 10^5^ HUVEC cells/cm^2^ were plated in the apical compartment, and the inserts were added to a 24-well plate. Following a culture period of 7 days, the Transwells® underwent treatment, and permeability studies were conducted [51]. During the maturation, TEER values were measured by epithelial volt/ohm (TEER) meter 3 (EVOM3) coupled with STX2 chopstick electrodes (World Precision Instruments, Sarasota, FL, USA) until reaching a TEER value ≥ 70 *Ω*cm^2^ before the stimulation [52, 53]. Cells were maintained for 15 min at 37°C and 5% CO_2_, and then the TEER values were measured again before starting the experiment to check the stability of the values. To assess the BBB-crossing capabilities of investigated drugs, they were introduced to the apical environment for 2–24 hr and detected by a 0.04% fluorescent tracer (Santa-Cruz, CA, USA). In particular, the medium at the bottom side of the Transwells® was used to measure the values of the PBS (Papp, cm/s) of green tea, saffron, trans-R, and citicoline® alone and in combination, and this was calculated with the following formula [54]:

(1) $$\begin{array}{c} \text{Papp}=dQ/dt⇥1/m0⇥1/A⇥\text{V Donor}. \end{array}$$

dQ: amount of substance transported (nmol or *μ*g);

dt: incubation time (sec);

m0: amount of substrate applied to donor compartment (nmol or *μ*g);

A: surface area of Transwell membrane (cm^2^);

VDonor: volume of the donor compartment (cm^3^).

Negative controls without cells were tested to exclude Transwell membrane influence.

### 2.6. Claudin 5 Assay Kit

The amount of claudin 5 in BBB cell lysates was analysed with the Claudin 5 ELISA Kit (MyBiosource, San Diego, CA, USA) according to the manufacturer's specifications. Cells were lysed using cold 1× PBS and then centrifuged at 1500 × *g* for 10 min at 4°C. After incubating 100 *μ*L of each sample at 37°C for 90 min, 100 *μ*L of detection solution A was added to each well and incubated for 45 min at 37°C. Post-washing, 100 *μ*L of detection solution B was applied to each well and incubated at 37°C for 45 min. After adding 90 *μ*L of substrate solution to each well, the plate was incubated at 37°C for 20 min in the dark. After stopping the reaction with 50 *μ*L of stop solution, the absorbance was analysed at 450 nm using a spectrometer (Infinite 200 Pro MPlex, Tecan, Männedorf, Switzerland). The concentration was expressed in ng/mL by comparing the data to the standard curve (0–2500 pg/mL) [55]. The data are presented as mean ± SD (%) compared to the untreated control sample.

### 2.7. Tricellulin (TRIC) Assay Kit

TRIC ELISA kit (MyBiosource, San Diego, CA, USA) was used to measure the TRIC/marveld in BBB cell lysates, following the manufacturer's instructions. After lysing cells with 1× cold PBS, they were centrifuged at 1500 × *g* for 10 min at 4°C. After incubating 100 *μ*L of each sample at 37°C for 90 min, the addition of 100 *μ*L of detection solution A was made and incubated for 45 min at 37°C. Post-washing, add 100 *μ*L of detection solution B to each well and incubate for 45 min at 37°C. Incubate the plate at 37°C for 20 min in the dark after adding 90 *μ*L of substrate solution to each well. Stop the reaction with 50 *μ*L of stop solution and analyse absorbance at 450 nm using a spectrometer (Infinite 200 Pro MPlex, Tecan, Männedorf, Switzerland). The data are compared to the standard curve (0.625–20 ng/mL) to determine concentration in ng/mL. The data are presented as mean ± SD (%) compared to the untreated control sample.

### 2.8. ROS Production

A standard procedure based on cytochrome C reduction on CCF-STTG1 cells quantified superoxide anion release [56]. After treatment, 100 *μ*L of cytochrome C (1 mg/mL, Merck Life Science, Rome, Italy) and 100 *μ*L of superoxide dismutase (0.1 mg/mL, Merck Life Science, Rome, Italy) were added to each sample for 30 min in an incubator. At 550 nm, a spectrophotometer (Infinite 200 Pro MPlex, Tecan, Männedorf, Switzerland) assessed absorbance in cell culture supernatants. O_2_ rate was represented as the mean SD (%) of nanomoles per decreased cytochrome C per microgram of protein relative to the control (0 line) [56].

### 2.9. NO Production

After stimulations, Griess' reagent (Promega, Italy) was added to CCF-STTG1/HUVEC cell supernatants to assess NO generation [57]. After stimulation, 50 *μ*L of supernatant was separated from each well and transferred to a fresh 96-well multiwell with 50 *μ*L of sulfanilamide solution. The plate was incubated at 37°C in the dark for 10 min. To finish, add 50 *μ*L of N-1-naphthyl ethylenediamine dihydrochloride (NED solution) to each well and incubate for another 10 min at 37°C in the dark. After incubation, a spectrophotometer reading (Infinite 200 Pro MPlex, Tecan, Männedorf, Switzerland) was recorded at 520–550 nm within 30 min. The assay detects NO^2−^, one of the most stable and least volatile NO derivatives. Results are percent (%) normalised to untreated samples, based on the standard nitrate calibration curve (0–100 *µ*M).

### 2.10. IL-2 Assay Kit

Following the manufacturer's instructions, Human IL-2 ELISA Kit (FineTest, Wuhan, China) was used to detect IL-2 in CCF-STTG1 cell lysates [58]. After adding 100 *µ*L of each sample to each well, the plate was incubated at 37°C for 90 min. After incubation, each well was cleaned twice with a wash buffer. Then, after adding 100 *µ*L of biotin-labeled antibody working solution to the wells, the plate was incubated at 37°C for 60 min. Post-incubation, wells were washed three times with wash buffer, and 100 *µ*L of SABC working solution was added. After incubating at 37°C for 30 min, the wells were cleaned, and 90 *µ*L of 3,3′, 5,5′-tetramethylbenzidine (TMB) substrate was added to each well. After 10–20 min, 50 *µ*L stop solution was added to each well, and the plate was read at 450 nm using a plate reader (Infinite 200 Pro MPlex, Tecan, Männedorf, Switzerland). Standard curves were drawn to correlate colour intensity (OD) with standard concentration (31.25–2,000 pg/mL). Results were presented as mean ± SD (%) vs. control (0 lines) from five independent experiments in triplicates.

### 2.11. TNF*α* Assay Kit

The plate was incubated at 37°C for 60 min after adding 100 *µ*L of biotin-labeled antibody working solution to the wells. After incubation, wells were washed three times with wash buffer, and 100 *µ*L of SABC working solution was added. Following a 30-min incubation at 37°C, each well was cleaned, and 90 *µ*L of 3,3′, 5,5′-Tetramethylbenzidine (TMB) substrate was added. After 10–20 min, 50 *µ*L stop solution was added to each well. The plate was read at 450 nm using a plate reader (Infinite 200 Pro MPlex, Tecan, Männedorf, Switzerland Colour intensity (OD) and standard concentration (31.25–2,000 pg/mL) were correlated using standard curves [47]. The five independent, triplicate tests' results were displayed as mean ± SD (%) against the control (0 lines).

### 2.12. BDNF Quantification ELISA Kit

Human BDNF ELISA Kit (Thermo ScientificTM, Waltham, MA, United States) quantified BDNF in CCF-STTG1 cell supernatants following the manufacturer's instructions. Each well received a biotinylated detection antibody, and the plate was incubated at room temperature for 1 hr. After 45 min of HRP-conjugated streptavidin incubation, TMB substrate solution was added for 30 min, and a stop solution terminated the reaction. To calculate BDNF concentration, absorbance was measured at 450 nm using a spectrometer (Infinite 200 Pro MPlex, Tecan, Männedorf, Switzerland) and compared to the standard curve (0.066–16 ng/mL) [49]. The data are presented as mean ± SD (%) compared to the untreated control sample. The cells were also given 10 ng/mL BDNF as a positive control [45].

Human BDNF Elisa Kit (Thermo ScientificTM, Waltham, MA, United States) quantified BDNF in CCF-STTG1 cell supernatants following the manufacturer's instructions. Each well received a biotinylated detection antibody, and the plate was incubated at room temperature for 1 hr. After 45 min of HRP-conjugated streptavidin incubation, TMB substrate solution was added for 30 min, and a stop solution terminated the reaction. To calculate BDNF concentration, absorbance was measured at 450 nm using a spectrometer (Infinite 200 Pro MPlex, Tecan, Männedorf, Switzerland) and compared to the standard curve (0.066–16 ng/mL) [49]. The data are presented as mean ± SD (%) compared to the untreated control sample. The cells were also given 10 ng/mL BDNF as a positive control [45].

### 2.13. p53 Assay Kit

Following manufacturer guidelines, p53 Transcription Factor assay kit (Cayman Chemical Company, Ann Arbour, MI, USA) was used to evaluate p53 level in CCF-STTG1 cell nuclear extracts. The cells were lysed with ice-cold Mix 1× complete hypotonic buffer with NP-40 and centrifuged at 12,000 × *g* at 4°C for 10 min. After solubilising the pellet with ice-cold complete nuclear extraction buffer 1x with protease and phosphatase inhibitors, the supernatant was centrifuged at 12,000 × *g* for 15 min at 4°C to measure p53 level and protein quantification using the bicinchoninic acid (BCA) assay (Thermo Fisher, Milan, Italy) [59]. The data are presented as mean ± SD (%) compared to the untreated control sample.

### 2.14. Cytochrome C Assay Kit

The cytochrome-C ELISA Kit (MyBiosource, San Diego, CA, USA) was used to measure the amount of cytochrome C in CCF-STTG1 cell lysates, following the manufacturer's instructions. The process involved adding 100 *μ*L of each sample to each well, incubating at 37°C for 90 min, then removing the material and adding 100 *μ*L of detection solution A for 45 min at 37°C. After incubation, wash the wells and add 100 *μ*L of detection solution B. Incubate for 45 min at 37°C. After adding 90 *μ*L of substrate solution, the plate was incubated at 37°C for 20 min in the dark. After stopping the reaction with 50 *μ*L of stop solution, the absorbance was measured at 450 nm using a spectrometer (Infinite 200 Pro MPlex, Tecan, Männedorf, Switzerland). The concentration was reported in ng/mL by comparing the data to the standard curve (15.6–500 nmol/L) [60]. The data are presented as mean ± SD (%) compared to the untreated control sample.

### 2.15. SIRT-1 Assay Kit

Quantification of SIRT1 protein was measured with SIRT1 ELISA Kit (Thermo ScientificTM, Waltham, MA, USA) on the CCF-STTG1 cell lysate. Add 100 *μ*L of each sample and incubate at 37°C for 90 min. Remove the material, add 100 *μ*L of detection solution A, and incubate for 45 min at 37°C. Following washing, 100 *μ*L of detection solution B was added to each well and incubated at 37°C for 45 min. After adding 90 *μ*L of substrate solution to each well, the plate was incubated at 37°C for 20 min in the dark. To stop the reaction, 50 *μ*L of stop solution was employed. A spectrometer (Infinite 200 Pro MPlex, Tecan, Männedorf, Switzerland) measured absorbance at 450 nm and expressed concentration as ng/mL against a standard curve (1.23–300 ng/mL) [61]. The data are presented as mean SD (%) vs. control (line 0).

### 2.16. Nrf2 Assay Kit

Nrf2 Quantification ELISA Kit (MyBiosource, San Diego, CA, USA) was used to measure the presence of Nrf2 in CCF-STTG1 cell lysates following the manufacturer's instructions. In summary, 100 *μ*L of each sample was incubated at 37°C for 90 min, removed, and washed three times in a 96-well plate. Next, 100 *μ*L of detection solution A was added and incubated at 37°C for 45 min. The wells were then washed, and 100 *μ*L of B was added. The plate was incubated at 37°C for 45 min. Incubate the plate at 37°C for 20 min in the dark after adding 90 *μ*L of substrate solution to each well. The reaction was stopped using 50 *μ*L of stop solution, and the absorbance was measured at 450 nm using a spectrometer (Infinite 200 Pro MPlex, Tecan, Männedorf, Switzerland). Comparing the findings to the standard curve (0.014 ng/mL–10 ng/mL) yields ng/mL [62]. Results are shown as mean SD (%) vs. control (line 0).

### 2.17. Amyloid Precursor Protein (APP) Assay Kit

The Amyloid Beta A4 protein ELISA Kit (Merck Life Science, Milan, Italy) measured the amount of APP on cellular supernatants of CCF-STTG1 cells following the manufacturer's instructions. After treatments, cellular supernatants were collected and analysed with an ELISA kit. Each well received biotinylated detection antibody for the target protein and the plate was incubated at room temperature for 1 hr. After 45 min of HRP-conjugated streptavidin incubation, TMB substrate solution was added for 30 min, and the reaction was halted with stop solution. To calculate APP concentration, absorbance was measured at 450 nm using a spectrometer (Infinite 200 Pro MPlex, Tecan, Männedorf, Switzerland) and compared to the standard curve (0.1–100 ng/mL) [44]. The data are presented as mean ± SD (%) compared to the untreated control sample.

### 2.18. Human Tau (Pospho) Protein Assay Kit

Tau (Pospho) protein was investigated by Tau (Phospho) [pS199] Human ELISA Kit (Thermo ScientificTM, Waltham, MA, USA) on CCF-STTG1 cell lysates. In summary, 100 *μ*L of each sample was added to the plate, incubated at 37°C for 90 min, removed, and washed three times. Next, add 100 *μ*L of detection solution A to each well and incubate at 37°C for 45 min. Post-incubation, wash wells, add 100 *μ*L detection solution B, and incubate at 37°C for 45 min. After adding 90 *μ*L of substrate solution, the plate was incubated at 37°C for 20 min in the dark. The reaction was stopped with 50 *μ*L of stop solution, and the absorbance was measured at 450 nm using a spectrometer (Infinite 200 Pro MPlex, Tecan, Männedorf, Switzerland). Results are shown as mean SD (%) relative to the control (line 0), and concentration is reported in ng/mL by comparing data to the standard curve (15.6–1,000 pg/mL) [63].

### 2.19. Statistical Analysis

The data were presented as mean SD (%) normalised to control values (0 lines) from at least five biological separate protocols with three technical replicates. Group comparisons were done using one-way ANOVA with Bonferroni's post hoc test or Mann–Whitney's *U* test in GraphPad Prism 5 (GraphPad Software, La Jolla, CA, USA). Statistical significance was determined at *p*  < 0.05.

## 3. Results

### 3.1. Effects on CCF-STTG1 Cell Viability of the Selected Extracts

**Figure 1:**
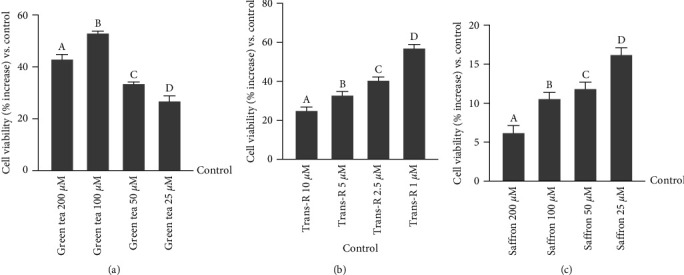
Dose-response study on cell viability in CCF-STTG1 cells tested by MTT test. (a) Dose-response study after green tea administration titrated 40% EGCG (200−25 *µ*M). The results are expressed as mean ± SD (%) of five independent experiments normalised to the control (0 lines corresponding to 100% cell viability), each performed in triplicate and expressed as the percentage increased. a, b, c and d *p*  < 0.05 vs. control; a is *p*  < 0.05 vs. c and d; b is *p*  < 0.05 vs. a, c and d; c is *p*  < 0.05 vs. d. (b) Dose-response study after trans-R administration (10−1 *µ*M). The results are expressed as mean ± SD (%) of five independent experiments normalised to the control (0 line corresponding to 100% cell viability), each performed in triplicate and expressed as the percentage increased. a, b, c and d *p*  < 0.05 vs. control; b is *p*  < 0.05*vs* a; c is *p*  < 0.05 vs. a and b; d is *p*  < 0.05 vs. a, b and c. (c) Dose-response study after saffron administration titrated 0.3% crocin (200−25 *µ*M). The results are expressed as mean ± SD (%) of five independent experiments normalised to the control (0 line corresponding to 100% cell viability), each performed in triplicate and expressed as the percentage increased. a, b, c and d *p*  < 0.05 vs. control; b and c are *p*  < 0.05 vs. a; d is *p*  < 0.05 vs. a, b and c.

After stimulation, the cell viability of CCF-STTG1 cells was assessed by MTT assays to determine the ideal concentration in a dose-response study of each extract without any cytotoxic effect after 24 hr of stimulation compared to the control (untreated sample). As reported in Figure 1(a), all green tea concentrations (ranging from 25 to 200 *µ*M) showed more beneficial effects than the control as reported by increased cell viability percentage (*p*  < 0.05); in particular, green tea 100 *μ*M appears to be the dose able to induce a higher result compared to the other concentrations (200 *µ*M about 33%, *p*  < 0.05; 50 *µ*M about 60% *p*  < 0.05; 25 *µ*M about 87%, *p*  < 0.05). At the same time, the effects of trans-R on CCF-STTG1 cells were assessed (Figure 1(b)). Within the range tested, trans-R 1 *µ*M exerted a greater beneficial effect on CCF-STTG1 cells than the control, as reported by increased cell viability percentage (*p*  < 0.05) and compared to the other concentrations 10 *µ*M, 5 *µ*M and 2.5 *µ*M (*p*  < 0.05, about 57%, 48%, and 31%, respectively). Finally, saffron was tested on CCF-STTG1 cells in a concentration range from 25 to 200 *µ*M (Figure 1(c)). Also, in this case, all concentrations showed no side effects on CCF-STTG1 cells, increasing their viability in percentage compared to the control (*p*  < 0.05) with a greater effect at 25 *µ*M, which increased cell viability percentage (*p*  < 0.05) by about 62.5%, 34%, and 20% compared to the other concentrations tested (200 *µ*M, 100 *µ*M, and 50 *µ*M, respectively). Based on these findings, the concentrations chosen for each extract are green tea 100 *μ*M, trans-R 1 *µ*M, and saffron 25 *µ*M, which are maintained for all successive experiments.

**Figure 2:**
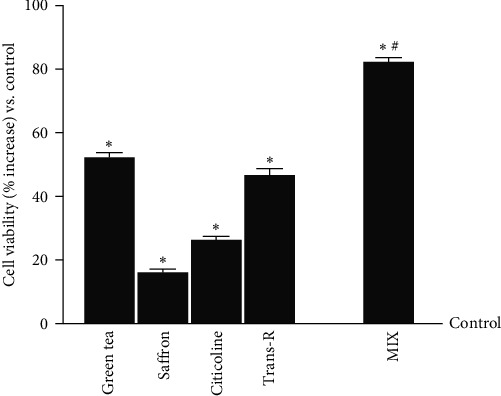
Cell viability on CCF-STTG1 cells measured by MTT test. Green tea titrated 40% EGCG = 100 *μ*M; saffron titrated 0.3% crocin = 25 *μ*M; citicoline = 100 *µ*M; trans-*R* = 1 *µ*M; MIX = green tea 100 *μ*M + saffron 25 *μ*M +citicoline 100 *µ*M + trans-R 1 *µ*M. The results are expressed as mean ± SD (%) of five independent experiments normalised to the control (0 line corresponding to 100% cell viability), each performed in triplicate and expressed as the percentage increased.  ^*∗*^*p* < 0.05 vs. control; ^#^*p* < 0.05 vs. green tea, saffron, citicoline, trans-R.

In addition to the dry extract already analysed, 100 *µ*M citicoline [43] has been investigated alone and combined with green tea 100 *μ*M, trans-R 1 *μ*M, and saffron 25 *μ*M. As reported in Figure 2, both the single agents and the combination tested (MIX) significantly increased cell viability compared to the control (*p*  < 0.05). In particular, the viability revealed that by combining the single agents, the observed effects are greater than green tea, saffron, citicoline, and trans-R alone (about 30%, 76%, 71%, and 43%, respectively), supporting the hypothesis that this combination can improve the neuronal functions.

### 3.2. Permeability and Integrity of BBB *In Vitro* Model

**Figure 3:**
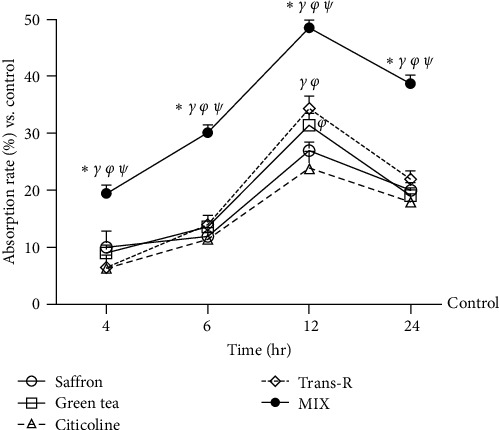
Permeability of the agents selected on BBB *in vitro* model. Absorption through BBB evaluated by fluorescent tracer. The abbreviations are the same as those used in Figure 2. Data are expressed as means ± SD (%) of five independent experiments normalised to control values (0% line), each performed in triplicate. All agents are *p*  < 0.05 vs. control;  ^*∗*^*p*  < 0.05 vs. green tea; ^*γ*^*p* <0.05 vs. saffron; ^*φ*^*p*  < 0.05 vs. citicoline; ^*ψ*^*p*  < 0.05 vs. trans-R.

Considering that the BBB is responsible for maintaining proper conditions of the CNS, further studies were conducted using a 3D *in vitro* model, simulating the complexity of the BBB *in vivo*, to evaluate the permeability and integrity of the barrier under stimulation conditions. For these reasons, the same agents used before were tested alone and combined at different time points (4-6-12-24 hr). As reported in Figure 3 and Table 1, the agents were able to cross the BBB with a maximum peak at 12 hr compared to the control (about 30% green tea 100 *µ*M, *p*  < 0.05; about 25% saffron 25 *µ*M, *p*  < 0.05; about 20% citicoline 100 *µ*M, *p*  < 0.05; about 35% trans-R 1 *µ*M, *p*  < 0.05), maintaining their effect until 24 hr after the stimulation. In addition, MIX was able to exert a greater effect in increasing the permeability compared to the single agents with a pick at 12 hr (about 33% vs. green tea 100 *µ*M, *p*  < 0.05; about 44% vs. saffron 25 *µ*M, *p*  < 0.05; about 55% vs. citicoline 100 *µ*M, *p*  < 0.05; about 22% vs. trans-R 1 *µ*M, *p*  < 0.05). Furthermore, the basolateral environment study indicated that MIX had a higher permeability rate than single agents (*p*  < 0.05), and the main effect was observed at 12 hr of stimulation (about 64% vs. green tea 100 *µ*M; about 55% vs. saffron 25 *µ*M; about 49% vs. citicoline 100 *µ*M, *p*  < 0.05; and about 79.6% vs. trans-R 1 *µ*M). All these findings support the hypothesis that MIX composed of trans-R 1 *µ*M, green tea 100 *µ*M, saffron 25 *µ*M, and citicoline 100 *µ*M can exert beneficial effects on CCF-STTG1 cells, which can use the agents to exert their neuronal functions.

**Figure 4:**
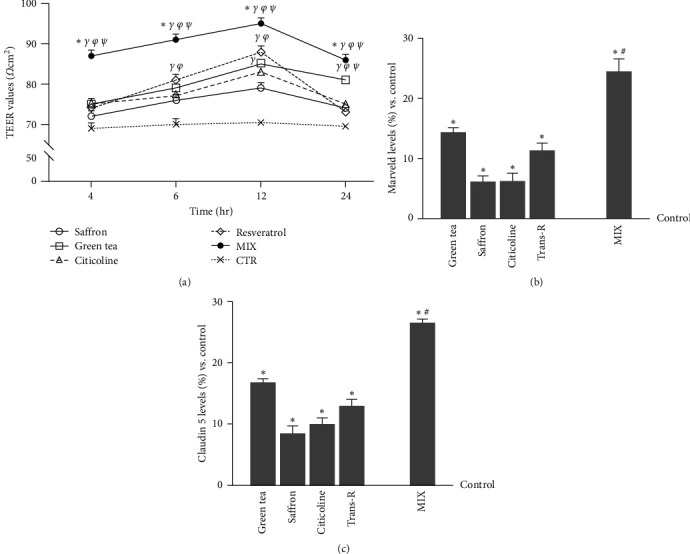
The integrity of BBB *in vitro* model. (a) the TEER values were measured using EVOM3. The breakpoint between the axes corresponds to the threshold value below which the experimental model does not mature. The abbreviations are the same as in Figure 2. The data are expressed as means ± SD (%) of five independent experiments performed in triplicates. All agents are *p* < 0.05 vs. control;  ^*∗*^*p* < 0.05 vs. green tea; ^*γ*^*p* < 0.05 vs. saffron; ^*φ*^*p* < 0.05 vs. citicoline; ^*ψ*^*p* < 0.05 vs. trans-R. (b) marveld and (c) claudin 5 levels were measured by the ELISA Kit under the same conditions as the TEER analysis. The abbreviations are the same as in Figure 2. The data are expressed as means ± SD (%) of five independent experiments normalised to control values (0% line), each performed in triplicates.  ^*∗*^*p* < 0.05 vs. control; ^#^*p* < 0.05 vs. green tea, saffron, citicoline, and trans-R.

The integrity of the BBB replicated *in vitro* was then confirmed through TEER evaluation and TJ analysis. TEER analysis (Figure 4(a)) showed that all single agents could maintain epithelial integrity, showing the peak in permeability at 12 hr. In addition, MIX was able to exert a greater effect at 12 hr compared to the control (*p*  < 0.05) and the single agents (about 10.5% vs. green tea 100 *µ*M, *p*  < 0.05; about 17% vs. saffron 25 *µ*M, *p*  < 0.05; about 13% vs. citicoline 100 *µ*M, *p*  < 0.05; about 8% vs. trans-R 1 *µ*M, *p*  < 0.05). To confirm the integrity of BBB, the TJs levels Figures 4(b) and 4(c) were explored. Notably, claudin-5, responsible for selectively decreasing ion permeability [39], and marveld protein, which maintains the barrier to macromolecule passage [41], were evaluated to prevent treatment-related changes. As reported in Figure 4(b), the data from the evaluation of claudin-5 level on the BBB showed a greater effect after the stimulation with MIX than the control (*p*  < 0.05) and all the single agents. It caused an increase of about 37% vs. green tea 100 *µ*M, about 68% vs. saffron 25 *µ*M, about 62% vs. citicoline 100 *µ*M and about 51% vs. trans-R 1 *µ*M (*p*  < 0.05). A similar effect was shown on the marveld level, in which MIX had a greater effect than the control and the single agents (Figure 4(c), about 41% vs. green tea 100 *µ*M, about 75% vs. saffron 25 *µ*M, about 74.5% vs. citicoline 100 *µ*M, and about 54% vs. trans-R 1 *µ*M, *p*  < 0.05).

### 3.3. Evaluation of Neuroprotective Effects of Selected Extracts under Oxidative Stress Conditions

**Figure 5:**
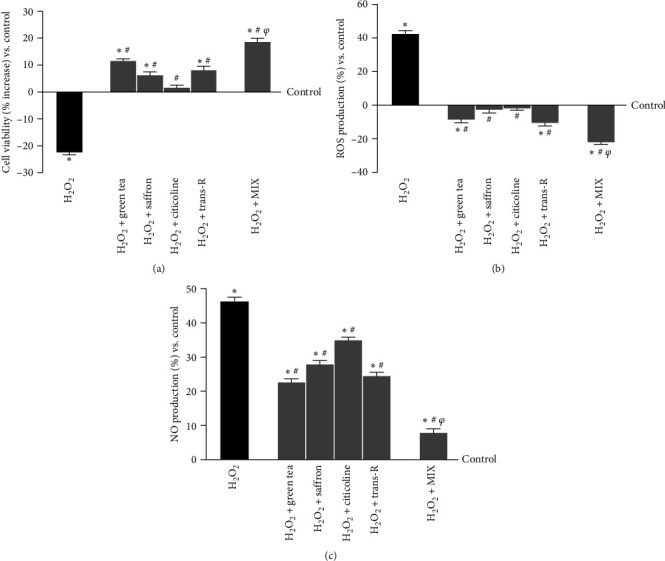
Analysis of the stimulation under oxidative stress conditions on CCF-STTG1 cells. (a) cell viability (percentage increased), (b) ROS production, and (c) NO production measured on CCF-STTG1 cells pretreated with H_2_O_2_ 200 *μ*M and then treated for 24 hr with the agents alone and MIX are illustrated. H_2_O_2_ = 200 *μ*M H_2_O_2_; H_2_O_2_ + green tea = 200 *μ*M H_2_O_2_ + green tea titrated 40% EGCG = 100 *μ*M; H_2_O_2_; H_2_O_2_ + saffron = 200 *μ*M H_2_O_2_ + saffron titrated 0.3% crocin = 25 *μ*M; H_2_O_2_; H_2_O_2_ + citicoline = 200 *μ*M H_2_O_2_ + 100 *µ*M citicoline; H_2_O_2_ + trans-R = 200 *μ*M H_2_O_2_ + 1 *µ*M trans-R; H_2_O_2_ + MIX = 200 *μ*M H_2_O_2_ + green tea 100 *μ*M + saffron 25 *μ*M + citicoline 100 *µ*M + trans-R 1 *µ*M. The results are expressed as mean ± SD (%) of 5 independent experiments performed in triplicates normalised to control (0% line).  ^*∗*^*p* < 0.05 vs. control; ^#^*p*  < 0.05 vs. H_2_O_2_; ^*φ*^*p*  < 0.05 vs. green tea, saffron, citicoline, and trans-R.

It is well known that brain antioxidant capability decreases with age, making the brain more susceptible to oxidative injury. In CCF-STTG1 cells, cell viability, ROS, and NO productions were measured to determine the selected extracts' cell repair capacity under oxidative conditions. As shown in Figure 5(a), the exposure to H_2_O_2_ 200 *μ*M significantly decreased cell viability (about 22% compared to the control, *p*  < 0.05), and it is reverted by the treatment with the agents alone and MIX (*p*  < 0.05). The most significant effect was obtained with MIX, which counteracts the cell loss. Indeed, MIX induced an increase in cell viability compared to the single agents (about 50% vs. green tea 100 *µ*M, *p*  < 0.05; about 60% vs. saffron 25 *µ*M, *p*  < 0.05; about 80% vs. citicoline 100 *µ*M, *p*  < 0.05; about 55% vs. trans-R 1 *µ*M, *p*  < 0.05). These data suggest that MIX could maintain cell viability more effectively than their use alone (*p*  < 0.05 vs. single agents). Further studies have been conducted on ROS production following the main theory behind brain ageing: green tea 100 *µ*M, saffron 25 *µ*M, citicoline 100 *µ*M, and trans-R 1 *µ*M alone and combined maintained low ROS levels (*p*  < 0.05 vs. control), indicating safety during use (Figure 5(b)). The exposure of CCF-STTG1 cells to H_2_O_2_ 200 *μ*M significantly increased the ROS production (about 42% compared to the control, *p*  < 0.05), which was statistically significantly counteracted by the stimulation with the single agents (about 50% green tea 100 *μ*M, about 40% saffron 25 *μ*M, about 37% citicoline 100 *μ*M and about 46% trans-R compared to H_2_O_2_ 200 *μ*M). At the same time, MIX amplified the reduction of ROS production compared to H_2_O_2_ 200 *μ*M and to the single agents (about 80% vs. H_2_O_2_ 200 *μ*M, *p*  < 0.05; about 60% vs. green tea 100 *μ*M, *p*  < 0.05; about 85% vs. saffron 25 *μ*M, *p*  < 0.05; about 87% vs. citicoline 100 *μ*M, *p*  < 0.05; about 50% vs. trans-R 1 *μ*M, *p*  < 0.05). These analyses demonstrate that the agents under investigation and MIX were able to reduce ROS production (*p*  < 0.05). Combined, they could exert a protective effect that positively influences the antioxidant capacity of neuronal cells by attempting to restore homeostasis, indicating that MIX has a neuroprotective effect. Since maintaining oxidative equilibrium is critical to avoid brain cell death, CCF-STTG1 cells activated under the same conditions were tested for NO generation. As shown in Figure 5(c), H_2_O_2_ 200 *µ*M increased NO production compared to the control (*p*  < 0.05), supporting the hypothesis of the cell loss previously observed through the viability and ROS production assay. The stimulation with the single alone and MIX counteracted the damaging action of H_2_O_2_ 200 *µ*M (*p*  < 0.05). In addition, the reduction observed in the presence of the MIX was also statistically significant compared to the single agents and H_2_O_2_ 200 *μ*M alone (about 89% vs. H_2_O_2_ 200 *μ*M, *p*  < 0.05; about 65% vs. green tea 100 *μ*M, *p*  < 0.05; about 72% vs. saffron 25 *μ*M, *p*  < 0.05; about 77% vs. citicoline 100 *μ*M, *p*  < 0.05; about 68% vs. trans-R 1 *μ*M, *p*  < 0.05). Analysis of NO generation shows its major involvement in neurodegenerative processes, generating highly reactive species to promote oxidative stress and cell death.

**Figure 6:**
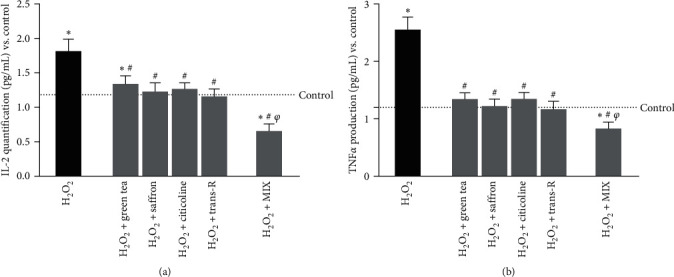
Analysis of the inflammatory panel on CCF-STTG1 cells under oxidative stress conditions. (a) IL-2 and (b) TNF*α* productions were measured on CCF-STTG1 pretreated with H_2_O_2_ 200 *μ*M and then treated for 24 hr with single agents alone and MIX. The results are expressed as mean ± SD (pg/mL) of 5 independent experiments performed in triplicate. The abbreviations are the same as those used in Figure 5.  ^*∗*^*p* < 0.05 vs. control; ^#^*p*  < 0.05 vs. H_2_O_2_; ^*φ*^*p*  < 0.05 vs. green tea, saffron, citicoline, and trans-R.

Since inflammation is another factor during brain ageing, IL-2 and TNF*α* productions were quantified (Figure 6). The analysis of inflammatory processes confirms the data obtained previously. Indeed, the agents examined were able to reduce inflammatory markers by decreasing the inflammation generated due to the induction of oxidative damage, as reported by decreased IL-2 and TNF*α* productions compared to H_2_O_2_-treated cells (*p*  < 0.05). MIX amplified the decrease of IL-2 production (Figure 6(a)) compared to the H_2_O_2_ 200 *μ*M and single agents (about 64% vs. H_2_O_2_ 200 *μ*M, *p*  < 0.05; about 51% vs. green tea 100 *μ*M, *p*  < 0.05; about 47% vs. saffron 25 *μ*M, *p*  < 0.05; about 48% vs. citicoline 100 *μ*M, *p*  < 0.05; about 43% vs. trans-R 1 *μ*M, *p*  < 0.05). Similarly, MIX was more effective in also decreasing TNF*α* production (Figure 6(b)) compared to the H_2_O_2_ 200 *μ*M and single agents (about 67% vs. H_2_O_2_ 200 *μ*M, *p*  < 0.05; about 38% vs. green tea 100 *μ*M, *p*  < 0.05; about 32% vs. saffron 25 *μ*M, *p*  < 0.05; about 38% vs. citicoline 100 *μ*M, *p*  < 0.05; about 29% vs. trans-R 1 *μ*M, *p*  < 0.05). These data suggest that the combination is more effective in decreasing the oxidative stress-related inflammatory pattern than the control (*p*  < 0.05) and the single agents (*p*  < 0.05), suggesting the synergic action of the MIX.

### 3.4. Study of Intracellular Pathways Activated by the Selected Extracts under Oxidative Stress

**Figure 7:**
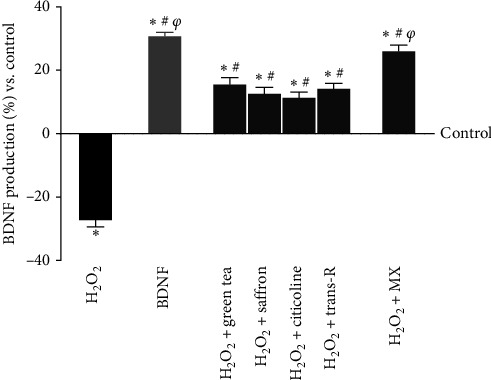
BDNF production under oxidative stress conditions. BDNF = 10 ng/mL. The abbreviations are the same as reported in Figure 5. The results are expressed as the mean ± SD (pg/mL) of five independent experiments performed in triplicate normalised to the control (0 line).  ^*∗*^*p* < 0.05 vs. control; ^#^*p*  < 0.05 vs. H_2_O_2_; ^*φ*^*p*  < 0.05 vs. green tea, saffron, citicoline, and trans-R.

In the study of cognitive decline, the analysis of BDNF production is also important, as it is a neurotrophin necessary for the survival of neurons, which can also interact with ROS, which is imbalanced in the mechanisms of ageing and neurodegenerative disease. Therefore, its presence was analysed following H_2_O_2_-induced damage and the stimulation with single agents and MIX. These data were also evaluated by comparing the treatment with 10 ng/mL BDNF, employed as a positive control. Again, all agents considered could stimulate BDNF production compared to the control, especially the damage induced (*p*  < 0.05). Indeed, as can be seen in Figure 7, oxidative stress dramatically reduces BDNF production (~30% vs. control, *p*  < 0.05), which comes to be counteracted by the treatment with the agents alone, bringing back the production to the control level (*p*  < 0.05). In addition, MIX significantly increases BDNF production compared to H_2_O_2_ 200 *μ*M and the single agents (about 2 times vs. H_2_O_2_ 200 *μ*M, *p*  < 0.05; about 40% vs. green tea 100 *μ*M, *p*  < 0.05; about 51% vs. saffron 25 *μ*M, *p*  < 0.05; about 56% vs. citicoline 100 *μ*M, *p*  < 0.05; about 45% vs trans-R 1 *μ*M, *p*  < 0.05) similar to what observed by 10 ng/mL BDNF (*p*  < 0.05). These findings suggest that MIX may activate the endogenous BDNF production systems, suggesting a possible trophic effect.

**Figure 8:**
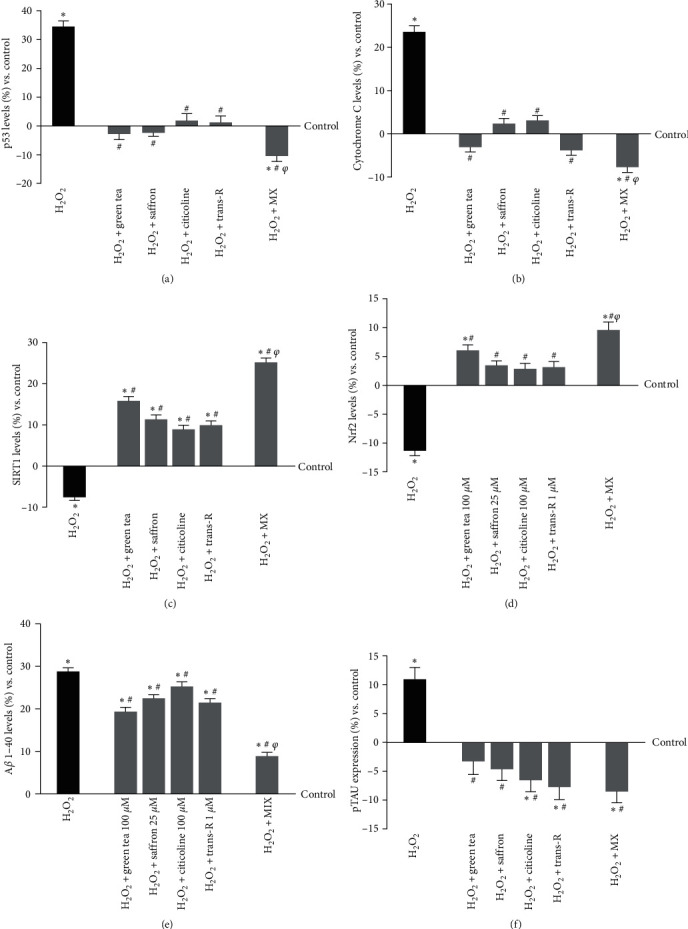
Analysis of biomarkers activity in CCF-STTG1 cells under oxidative stress condition. (a) p53, (b) cytochrome C (c), SIRT-1, (d) Nrf2, (e) A*β* 1-40, and (f) pTAU protein were reported. The tests were performed on CCF-STTG1 cells pretreated with H_2_O_2_ 200 *μ*M and then treated for 24 hr with single agents alone and MIX. The abbreviations are the same as those used in Figure 5. The results are expressed as mean ± SD (%) of five independent experiments normalised to control, each performed in triplicate.  ^*∗*^*p*  < 0.05 vs. control;  ^*#*^*p*  < 0.05 vs. H_2_O_2_; ^*φ*^*p*  < 0.05 vs. green tea, saffron, citicoline, and trans-R.

The apoptosis cascade is the fundamental mechanism of neuron cell death. Hence, research was done to find indicators. Under oxidative stress, CCF-STTG1 cells were examined for p53, a critical factor in ageing, oxidative stress, neurodegeneration, and cytochrome C, a key regulator of energy metabolism and apoptosis. Data reported in Figure 8(a) showed a reduction in p53 level after stimulation with all agents alone and in combination compared to the control (*p*  < 0.05) in cells pretreated with H_2_O_2_ 200 *μ*M. MIX amplified the reduction compared to H_2_O_2_ 200 *μ*M (~10.5%, *p*  < 0.05) and the single agents (about 73% vs. green tea 100 *μ*M, *p*  < 0.05; about 75.6% vs. saffron 25 *μ*M, *p*  < 0.05; about 87% vs. citicoline 100 *μ*M, *p*  < 0.05; about 81% vs. trans-R 1 *μ*M, *p*  < 0.05), indicating possible survival favoured conditions. The involvement of the cytochrome C analysed under the same conditions (Figures 8(b)) showed similar results, confirming the previously observed beneficial effects. The combination maintained basal cytochrome C, preserving mitochondrial integrity. In contrast, cells treated with H_2_O_2_ alone showed an increase in cytochrome C level that was significantly decreased by subsequent stimulation with the single agents and MIX (*p*  < 0.05, about 59% vs. green tea 100 *μ*M, *p*  < 0.05; about 70% vs. saffron 25 *μ*M, *p*  < 0.05; about 60% vs. citicoline 100 *μ*M, *p*  < 0.05; about 50% vs. trans-R 1 *μ*M, *p*  < 0.05). In addition, the level of SIRT1, which is critical for the control of energy metabolism through the gluconeogenic/glycolytic pathways via the PGC−1*α*/Nrf2 pathway, leading to increased mitochondrial function, was further investigated. As can be seen in Figures 8(c) and 8(d), all single agents and MIX improve both parameters, supporting the above observation (*p*  < 0.05); in particular, MIX generates a greater effect on both markers than single agents by increasing the expression of SIRT−1 of about 37% vs. green tea 100 *μ*M, about 55% vs. saffron 25 *μ*M, about 65% vs. citicoline 100 *μ*M, about 61% vs. trans-R 1 *μ*M (*p*  < 0.05) and for Nrf2 about 37% vs. green tea 100 *μ*M, about 64% vs. saffron 25 *μ*M, about 70% vs. citicoline 100 *μ*M and about 67% vs. trans-R 1 *μ*M (*p*  < 0.05). Finally, amyloid beta 1–40 peptide (A*β* 1–40) and pTau expression were also analysed to investigate the mechanisms activated in the neurodegeneration process Figures 8(e) and 8(f). Regarding A*β* 1–40, the previously observed data about the beneficial effect on brain trophism are confirmed; indeed, MIX amplifies the beneficial effect compared to the single agents (about 54% vs. green tea 100 *μ*M, *p*  < 0.05; about 61% vs. saffron 25 *μ*M, *p*  < 0.05; about 65% vs. citicoline 100 *μ*M, *p*  < 0.05; about 59% vs. trans-R 1 *μ*M, *p*  < 0.05). Similarly, data obtained from the analysis of pTau support the hypothesis of prevention of cognitive impairment. Again, MIX was able to induce significant improvement over H_2_O_2_-induced impairment compared to single agents (about 61% vs. green tea 100 *μ*M, *p*  < 0.05; about 45% vs. saffron 25 *μ*M, *p*  < 0.05; about 23% vs. citicoline 100 *μ*M, *p*  < 0.05; about 10% vs. trans-R 1 *μ*M, *p*  < 0.05).

## 4. Discussion

Life expectancy has been increasing, leading to a rise in the average age of the global population. Unfortunately, neurodegenerative diseases, including dementia, increase with age because the brain becomes more susceptible to antioxidant defence system imbalance, resulting in increased oxidative stress and a gradual decline in physiological functions [44]. There are 50 million persons with dementia worldwide, and this number is anticipated to rise by 150% in 25 years [64, 65]. Neurodegenerative disorders are currently defined as chronic and incurable conditions that cause temporary or permanent disabling effects in the affected person over time [49, 66]. Pharmacological therapies available for cognitive decline and dementia are not effective in reversing brain damage or halting disease progression but only in slowing the decline [67]. Since ageing cannot be stopped or eliminated, the discovery of new strategies for preventing and counteracting this process is required. It has been demonstrated that modifying one's lifestyle can reduce the likelihood of acquiring dementia, with diet being a key factor [68, 69]. Several studies are currently investigating natural substances with biological effects, particularly antioxidants, for use as a first-line therapy in the early stages of the disease or in combination with drugs in more advanced stages [7]. One option for intervention to lessen the physiological and pathological process of cognitive decline is using nutraceuticals with antioxidant and anti-inflammatory action, modifying mitochondrial stress, apoptotic factors, free radical scavenging system, and neurotrophic factors [7]. It is well recognised that the brain produces excessive ROS due to its high oxygen consumption, low levels of helpful antioxidant enzymes, and low catalytic activity. Among the most studied substances with antioxidant properties are green tea, saffron, trans-R, and citicoline. Indeed, green tea dry extract contains great quantities of polyphenols, most of which are flavanols (such as catechins, ECs, and procyanidins) and phenolic acids (such as hydroxybenzoic acid and hydroxycinnamic acid) [12]. In particular, green tea catechins may avoid age-related neurodegeneration by promoting natural antioxidant defence systems, modifying brain growth factors, reducing the neuroinflammatory pathway, and controlling apoptosis [13]. In addition, many studies have focused on exploring the effects of Saffron, which have revealed its numerous biological activities on human health, including its anti-tumour [70, 71], antidepressant, anti-anxiety [72, 73], antioxidant, anti-inflammatory [74, 75], and antinociceptive properties [76, 77]. Resveratrol may reduce oxidative stress by inhibiting genes coding for prooxidant proteins, activating genes coding for different antioxidant enzymes, and enhancing the expression of several memory-related proteins [78, 79]. In addition, it acts by enhancing glial, oxidative, and inflammatory responses via increasing the expression of heme oxygenase-1 (HO1); it has also been demonstrated that resveratrol may upregulate the HO1 expression by activating erythroid nuclear factor 2-related factor 2 (Nrf2) [80]. Finally, the neuroprotective action of citicoline was investigated in many preclinical studies. In an *in vivo* study, it has been demonstrated that this molecule can decrease the BBB permeability following damage and increase the activity of antioxidant defences while reducing prooxidant species levels [81]. All these agents have shown evidence of improving antioxidant defence and addressing symptoms associated with ageing and cognitive decline, such as anxiety, depression, and insomnia. For the first time, the present research showed how combining these four active ingredients could synergistically impact CCF-STTG1 cell activity, suggesting a potential new tactic to halt neurodegenerative processes and delay cognitive decline. First, it was ensured that the agents tested at physiological concentrations reached the target site and crossed the BBB. For this reason, a model was developed to mimic the BBB structure and investigate the passage of the single compounds and the MIX, through the analysis of claudin-5 and marveld proteins tests. This allowed us to demonstrate that MIX can cross the BBB more efficiently than single compounds while maintaining its integrity. This led to the hypothesis of its safety and activity at the CNS level, where it could act for up to 24 hr without altering the permeability flux.

Subsequently, the effects of the single compounds and the MIX in the presence of an oxidative stimulus provided by hydrogen peroxide were investigated. It has been shown that, while the single compounds have an antioxidant activity by improving mitochondrial metabolism and decreasing ROS and NO production when combined in the MIX, they demonstrate greater effectiveness and synergistic effect. Furthermore, by investigating the upstream expression of the transcription factor Nfr2, which regulates the production of antioxidant key molecules within the cell, it was possible to demonstrate a potential mechanism of action through which MIX effectively reduces ROS and NO downstream [82]. Moreover, even in this case, MIX was more effective than the single active compounds in increasing the expression of Nrf2. Therefore, we can hypothesise that the antioxidant effect is exerted upstream at the level of the transcription factor. Since oxidative stress is closely linked to inflammation as the two processes tend to influence each other [83], subsequent experiments focused on the effects of the single agents and the MIX on the marker of inflammation associated with cognitive decline. The results showed that the single compounds exert anti-inflammatory activity, but the MIX had a more significant impact on reducing inflammation levels. Therefore, simultaneously stopping oxidative stress and inflammation, the MIX may counteract that vicious circle that leads to neurodegeneration.

A member of the sirtuin family, SIRT1 is a class III NAD^+^-dependent histone deacetylase enzyme. Seven different sirtuin types (SIRT1–7) exist. The brain, heart, liver, kidneys, pancreas, skeletal muscles, spleen, and white adipose tissue are the organs where SIRT1 is highly expressed. SIRT1 is a key component of neural plasticity and cognitive development in the brain and is mostly found in the nuclei of neurons in the hippocampus, thalamus, and solitary tract. Cao et al. [24] found that trans-R can boost cognitive function by targeting SIRT1, specifically by acting on the antioxidant system, inflammatory system, cerebral blood flow, and synaptic plasticity [24]. As regards the latter, this occurs because SIRT1 can regulate the expression of BDNF [24]. Therefore, the effect of the single active agents and the MIX on SIRT1 was studied. Results demonstrated that the MIX could counteract, in a synergistic manner compared to the single compounds, the decrease in SIRT1 due to oxidative stress, thus demonstrating its strong neuroprotective activity.

BDNF is a neurotrophin involved in neuronal renewal and maintenance of cerebral blood flow, also contributing to neuroprotection [25]. Wicincki et al. [25] demonstrated the beneficial effects of trans-R on BDNF. Based on this evidence, it was investigated whether MIX could also increase BDNF levels and thus exert neuroprotective activity by producing this neurotrophin. In confirmation of what has already been demonstrated for the other markers, the MIX has been shown to have a positive and synergistic effect compared to the single compounds on the BDNF level.

The p53 protein, well-known for its roles in tumour suppression and DNA repair, also has implications in neurodegenerative processes. Research has shown that p53 can influence various aspects of neurodegeneration through its involvement in apoptosis, oxidative stress response, and cellular senescence. p53 may play a role in promoting neuronal cell death, which is a hallmark of neurodegeneration. Additionally, p53 regulates the expression of genes associated with oxidative stress and mitochondrial dysfunction, which are common features of neurodegenerative disorders. Furthermore, p53′s ability to induce cellular senescence has been linked to age-related neurodegenerative changes [84]. The results demonstrated that MIX positively modulates the p53 level to create a favourable condition for survival more efficiently than the single compounds.

Additionally, the role of cytochrome C was examined as a crucial controller of both cellular energy metabolism and apoptosis in astrocytes. Degenerative disorders are often accompanied by oxidative stress and mitochondrial malfunction. These conditions can cause an increase in membrane permeability and the release of cytochrome C, which in turn contributes to neurodegeneration [85]. MIX can decrease the amounts of cytochrome C during oxidative damage, promoting the maintenance of mitochondrial integrity.

We finally wanted to investigate the expression of two characteristic markers of dementia, particularly Alzheimer's disease, which is the most common among dementias. For this purpose, we analysed the A*β* 1–40 peptide and the expression of pTau protein. Concerning A*β* 1–40, the MIX exhibits greater inhibitory activity than the single active compounds in inhibiting its formation. As for pTau, results indicated that trans-R inhibits its expression, similar to the MIX.

## 5. Conclusions

Overall, this work demonstrates that a combination of green tea, saffron, trans-R, and citicoline, known as MIX, can potentially effectively treat CCF-STTG1 cells experiencing oxidative stress. This suggests there may be opportunities to create novel approaches for addressing brain ageing at various stages. Furthermore, MIX mitigates the detrimental impacts of the mechanisms associated with neurodegenerative disorders, indicating the potential for creating a novel formulation that can decelerate the intracellular processes linked to cognitive decline and dementia. This study supports the creation of a new nutraceutical supplement that may ameliorate the human brain's ageing condition. Further studies involving patients are encouraged to validate the formulation's effectiveness in clinical practice.

## Figures and Tables

**Table 1:** Permeability values of the agents selected on BBB *in vitro* model. Data < 0.2 × 10^−6^ cm/s indicates inadequate absorption with bioavailability < 1%, data between 0.2 and 2 × 10^−6^ cm/s indicates 1–90% bioavailability, and data > 2 × 10^−6^ cm/s shows over 90% bioavailability.

Substances	Time			
	4 hr	6 hr	12 hr	24 hr
*φ* Green tea	0.22 x 10^−6^	0.33 x 10^−6^	0.76 x 10^−6^	0.46 x 10^−6^
Saffron	0.24 x 10^−6^	0.29 x 10^−6^	0.65 x 10^−6^	0.48 x 10^−6^
Citicoline	0.2 x 10^−6^	0.28 x 10^−6^	0.58 x 10^−6^	0.44 x 10^−6^
*γ φ* Trans-R	0.2 x 10^−6^	0.34 x 10^−6^	0.83 x 10^−6^	0.53 x 10^−6^
^*∗*^*γ φ ψ* MIX	0.47 x 10^−6^	0.72 x 10^−6^	1.18 x 10^−6^	0.94 x 10^−6^

The abbreviations are the same as those used in Figure 2. All agents are *p*  < 0.05 vs. control;  ^*∗*^*p*  < 0.05 vs. green tea; ^*γ*^*p* < 0.05 vs. saffron; ^*φ*^*p*  < 0.05 vs. citicoline; ^*ψ*^*p*  < 0.05 vs. trans-R.

## Data Availability

All the data used to support this study's findings are available from the corresponding author upon reasonable request.

## Abbreviations

ADAM17: A disintegrin and metalloproteinase domain 17
A*β*1–40: *β*-amyloid peptide 1–40
BBB: Blood-brain barrier
BCA: Bicinchoninic acid
BDNF: Brain-derived neurotrophic factor
CDP: Citicoline or cytidine diphosphate-choline
CNS: Central nervous system
COX-2: Cyclooxygenase-2
DMEM: Dulbecco's modified eagle medium
ECG: Epicatechin-3-gallate
EGC: Epigallocatechin
EGCG: Epigallocatechin-3-gallate
EVOM3: Epithelial volt/ohm (TEER) meter 3
FBS: Foetal bovine serum
GSK-3*β*: Glycogen synthase kinase 3*β*
HO1: Heme oxygenase-1
HUVEC: Human umbilical vein endothelial cells
IL-1*β*: Interleukin-1 *β*
IL-2: Interleukin-2
IL-6: Interleukin-6
IL-10: Interleukin-10
MCP-1: Monocyte chemoattractant protein-1
MTT: 3-(4,5-Dimethylthiazol-2-yl)-2,5-diphenyltetrazolium bromide
NF-kB: Nuclear factor kappa-light-chain-enhancer of activated B cells
NO: Nitrogen oxide
NP-40: Nonionic polyoxyethylene surfactant 40
Nrf2: Erythroid nuclear factor 2-related factor 2
P/S: Penicillin–streptomycin
Papp: Apparent permeability coefficient
PBS: Phosphate-buffered saline
RANTES: Regulated upon activation, normal T cell expressed and presumably secreted
ROS: Reactive oxygen species
RPMI: Roswell Park Memorial Institute medium
SIRT1: Sirtuin 1
TEER: Transepithelial electrical resistance
TJ: Tight junction
TMB: 3,3′,5,5′-Tetramethylbenzidine
TNF*α*: Tumor necrosis factor alpha
TRIC: Tricelluli.
